# The Reductio Ad Absurdum of “Christian Science”

**Published:** 1899-01

**Authors:** 


### The Reductio Ad Absurdum of "Christian Science."

The *British Medical Journal* for November 12th, in an article entitled "Christian Science: What It Is," cites, with comments, several of the leading propositions of Mrs. Eddy's puerile cult: Matter does not exist. It is only a false conception of the human mind. Nothing bad exists. It is only "thinking makes it so," as Hamlet says. Disease does not exist. It, too, is merely a false conception of the mind, whence it follows *per contra* that health must be the same. Pain, and even death, are delusions which must be exorcised from the mind by means of the inculcation of "Truth." That being the case, of course vigor and life must be non-existent likewise. All this wonderful philosophy is contained in a book by the Rev. Mary Baker Eddy, entitled *Science and Health with a Key to the Scriptures.* We have carefully perused this book, and are irresistibly reminded of another of Shakespeare's inspired remarks: "The devil can cite Scripture for his purpose."

The *British Medical Journal* quotes from Mrs. Eddy's book the following passages: "The metaphysics of Christian science, like the rules of mathematics, prove the rule by inversion. For example: There is no pain in Truth and no truth in pain; no nerve in Mind and no mind in nerve; no matter in Mind and no mind in matter; no matter in Life and no life in matter; no matter in Good and no good in matter." Again, "A hypodermic injection of morphine is administered to a patient and in twenty minutes the sufferer is quietly asleep. To him there is no longer any pain. Yet any physician—allopathic, homœpathic, botanic, eclectic—will tell you that the troublesome material cause is unremoved, and that in a few hours, when the soporific influence of the opium is exhausted, the patient will find himself in the same pain unless the belief which occasions the pain has meanwhile disappeared. Where is the pain while the patient sleeps?" On this the *Journal* remaks: "This conundrum we must leave to the Christian scientists." We do not consider the conundrum so difficult of answer. Passing by the untrue assertion that any educated physician of any school would assert that pain will return unless the *belief* which occasions the pain has disappeared, we would point out that pain is a sensation, and is therefore dependent upon an act of perception, not of belief. True, by a sort of "short-circuiting" cerebral process a false conception of pain, seemingly peripherally originated, may really originate centrally, as occurs in hysteria, or in the hallucinations of the senses found in delirium. No one supposes pain to be material though it is a function or property of matter, just as weight is not material, though it can not be conceived of by the mind until mentally associated with matter. We can not see motion in the abstract, but we can recognize its existence through its effect upon matter.

The action of the morphine is to deaden this sense of perception whereby the mind becomes conscious of unwonted forces, or normal forces operating in an unwonted manner in the body. When the "Christian scientist" therefore asks Where is the pain? he is talking nonsense. The abnormal forces are at work just the same, but he is prevented by the morphine, which chains up his powers of perception, from perceiving them, just as a man chained to the wall in an absolutely dark room would be unable to perceive a pendulum swinging noiselessly from the ceiling out of his reach.

Or, take another example: An operator at one end of a telegraph circuit sends a message; it is conducted along the wire and signals the message on the instrument at the other end. But that instrument gets out of order, or the receiving operator falls asleep. Where is the message? asks the "Christian scientist." Surely it is just as much in existence as though the instrument were in order or the operator awake.

To a certain degree and under certain circumstances "suggestion," whether hypnotic or otherwise, has a similar effect. We can see the influences of suggestion operating all through life, and, as has been aptly said, "Our lives are but the reflex of the suggestions around us."

It has been said in theological science that every heresy that survives does so solely in virtue of the grain of truth that it enshrines, and the same is true of physical science. The grain of truth in "Christian science" is the influence of "suggestion" on the human economy; and it is to suggestion solely that whatever good results are in occasional instances attained by "Christian science" methods are due. That such are occasionally so attained is no doubt true, and it is to this fact that the adherence of a few perfectly conscientious and upright people to this eminently commercial cult is to be attributed.

But the applications of suggestion are strictly limited to a certain class of cases, and, moreover, it is in the highest degree advisable, indeed, imperative, that the use of even scientific suggestive therapeutics should be confined to properly trained persons, who are competent to recognize the nature of and to deal radically with the morbid processes actually at work, and to which the mere phenomena which suggestion can affect are attributable. A great deal of harm can already be traced to the injudicious use of suggestive therapeutics at the hands of persons unpossessed of the scientific training requisite to make them competent to use it judiciously.

Moreover, while the physician merely uses suggestion, or gives morphine in organic disease to procure temporary symptomatic relief, he does not rely on it to cure, but has recourse subsequently to radical measures for removing the cause where possible; and it is in placing obstacles in the way of the use of such measures that the "Christian science" folly is morally criminal.

In therapeutic suggestion, the great art lies in the ability to present the suggestion in such a persuasive, convincing, and apparently probable light as to command the assent of the subject. It is just here that whatever success "Christian scientists" occasionally attain is to be found, and for this reason man's nature is such that he instinctively inclines toward credence in the possibility of anything that claims to be based upon the omnipotent attributes of a Deity. This claim brings the mind to a state of passive receptivity, since most men believing in a Deity, who must be *ex hypothesi* omnipotent, are prepared to assent to the proposition that there is nothing that is not *possible* for him to effect. But it is also *possible* for him equally to keep man alive without the material elements of food or drink, or in an atmosphere destitute of oxygen; to keep him warm without clothing or shelter, and to reproduce his race without the mediation of the act of material congress. Yet he does not usually do so, and we have still to hear of the "Christian scientist" who carries his or her contempt for that delusion, matter, to the extent of abstaining altogether from food, drink, clothing, and house shelter, or who proposes to abolish the relation of the sexes. Moreover, the avidity with which the "Christian scientist" absorbs that eminently material product, money, is by no means assuring to persons of common sense in the light of his denunciation of the falsity of the "material," and his assertion that only the spiritual is real. Why does not the adherent of this nineteenth century bubble recognize that the other material things of this life, as well as the money wherewith to purchase them, are also "mere substitutes for the dignity and potency of the Divine Mind and its power" to sustain life, and as such condemn and discard them as he does material aids to healing?

That he does not is evidenced not only by the enormous fees charged for pupilage in this college of quackery, and extorted for its ministrations from its dupes, but it is shown by a case recorded in the New York *Times* for November 25th, which would be ludicrous were it not so pitiful an example of the depravity of human nature. A man who asserted that he had been injured by falling down an unprotected areaway, and was cured of his injuries by a "Christian science" healer, brought suit in the District Court of Des Moines, Iowa, to recover damages for his alleged injuries. Inasmuch as the process of healing consisted in the inculcation of the "Truth" that he had not really sustained any injury at all, but that his sensations on this point were merely delusions of his mind, the man was undoubtedly morally guilty of an atttempt to perpetrate a fraud. Fortunately, the judiciary had sufficient sense and courage to throw the case out on the ground that "injuries which could be cured by 'Christian science' must have been either wholly imaginary, or so nearly so that their estimation in dollars and cents, and even in cents alone, was impossible."

The *British Medical Journal* further points out that from a "Christian science" point of view medical treatment is not merely useless but immoral, and it quotes in support of this position the following paragraph, again from Mrs. Eddy's jumble of nonsense and irreverence: "Drugs, cataplasms, and whiskey are stupid substitutes for the dignity and potency of the Divine Mind and its power to heal. It is pitiful to lead men into temptation through the by-ways of physiology and materia medica, to victimize the race with intoxicating prescriptions for the sick, until mortal mind acquires an educated appetite for strong drinks, and men and women are made loathsome sots." What logic is here? Pain and disease do not exist; they are only hallucinations, because they are manifestations in the material plane, which material plane has no real existence, but is purely illusory. How, then, can the physical vices of alcoholism, morphïnism, and sottishness exist, for they are the productions of the material also, and must therefore be equally illusory? How can physical unchastity exist, since it is a result of material contact? The drunken man is not drunk, because the condition of alcoholism is the effect of material substance upon his material body, and matter is illusory. Perhaps it will be asserted that this is the exception which proves the rule, on the ground that alcohol is spirit; which would be really no more absurd than most of their arguments.

Well, then, since disease is a delusion, a patient does not really contract syphilis as the result of an impure connection, and therefore there is nothing for him to be ashamed of in being possessed of the delusion that he has. Neither is it possible for him to communicate it; he can only give rise to a delusion. What sense, therefore, is there in interdicting intercourse to syphilitics? Further, the physical act whereby he thinks he contracted the disease is not real, being material, therefore he is not guilty of unchastity.

In short, this pernicious folly strikes at the very root of all purity and morality, as much as it makes of life and responsibility a farce, and of all religious feeling of whatever kind a travesty.

In conclusion, the whole farrago of nonsense reminds us very strongly of one verse of a student's song on the various philosophic systems of the day, which runs somewhat as follows:

We can't assume, so Comte affirms, a first or final cause, sir,
Phenomena are all we know, their order and their laws, sir;
While Hegel's modest formula, a single line to sum in,
Is nothing is, and nothing's not, but every thing's becoming,
With a bow, wow, wow, etc.

—*Editorial in New York Medical Journal.*

